# Diphenyl Dimethyl Bicarboxylate in the Treatment of Viral Hepatitis, Adjuvant or Curative?

**DOI:** 10.4021/gr2008.10.1231

**Published:** 2008-11-20

**Authors:** Chen Wang, You Qing Xu

**Affiliations:** a Department of Gastroenterology, Beijing Tiantan Hospital, Capital Medical University, Beijing, 100050, China

**Keywords:** schisandra chinensis, schisandrin B, dimethyl diphenyl bicarboxylate, adjuvant, liver disease, viral hepatitis

## Abstract

Diphenyl dimethyl bicarboxylate (DDB) has been used in some countries as hepatoprotectant adjuvant in the treatment of liver diseases, such as chronic viral hepatitis, chemical or drug induced hepatic damage. Its early confirmed efficacy is to normalize elevated blood alanine aminotransferase (ALT) from different etiologies, however, it can rarely affect the rest hepatic enzymes. In addition, the lowering or normalization of ALT in most cases occurs during DDB treatment, withdrawal of DDB administration results in ALT re-elevated. Hence, for a long time, it has been only used as adjuvant of liver disease therapy. It is still controversial that whether DDB can be beneficial to liver histology. The normalization of ALT in hepatitis does not indicate therapeutic efficacy if without substantial liver histology improvement. In recent years, more studies showed that DDB might have new therapeutical potentials in liver diseases, it may have the effect of anti-viral, anti-malignancy. These new findings were mostly based on the in vitro or animal experiments, more basic studies and clinical trials are needed to ascertain these efficacies, prior to that stage, it is recommended to be cautious to apply DDB clinically for anti-virus and anti-malignancy purposes.

## Introduction

From the ancient time, the Chinese herb schisandra chinensis has been used as bactericidal agent in some Asian countries(1). From time to time, it is indicated in cases of chronic cough and dyspnea, diarrhea, night sweats, wasting disorders, irritability, palpitations, dream-disturbed sleep and insomnia(2).

The main active constituents of schisandra chinensis related to liver disease therapy are schisandrin B and schisandrin C. Diphenyl dimethyl bicarboxylate (DDB) is a synthesized intermediate derivative of schizandrin C. DDB is commonly used as adjuvant hepatoprotectant in the treatment of chronic viral hepatitis and other liver diseases with different etiologies in China(3) and some other Asian countries(4-6). Only in recent decades, the world began to understand its pharmacological possibilities and clinical applications(7). In recent years, people are enthusiastic to excavate its new therapeutic potentials in the treatment of liver diseases, and indeed, new pharmacological links to the liver diseases have been found. Along with these progresses, there are also certain controversies about this synthetic compound. In the past, people usually used it as an agent for improving liver function and lowering serum alanine aminotransferase (ALT) in viral hepatitis and in liver injuries induced by various chemicals and drugs(8-10). However, people recently started to investigate its effect in anti-virus, treating fatty liver, and anti-malignancy. Is schisandra chinensis born to be a completely liver herb? Can DDB play a principal role in the treatment of viral hepatitis and other liver damages? In this concise review, we focus on the current findings of DDB in the treatment of liver disease, and discuss the novel possibilities of DDB for further roles in liver disease therapy.

### Compound characteristics

**Figure 1:**
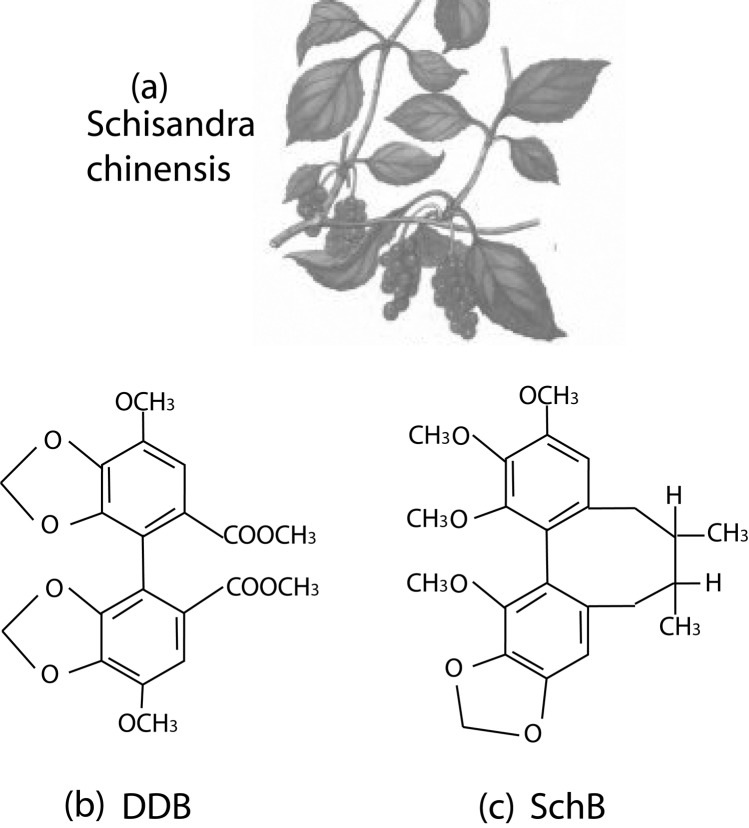
The schisandra chinensis, and the molecular structures of diphenyl dimethyl bicarboxylate (DDB), schisandrin B (Sch B).

Schisandra chinensis is a deciduous woody climbing vine about eight meters long which produces red spherical fruit. Because the whole fruit (including seeds) is said to have a salty taste, the skin and pulp are sweet and sour, and the kernels are pungent and bitter, therefore its name in Chinese is “Wu Wei Zi” denoting “five flavors”(Fig. 1a).

Schisandra fruit contains dibenzocyclooctene lignans (about 2% by weight), with the main constituents being schisandrin, schisandrin A, schisandrin B, schisandrin C, g-schisandrin (the racemic form of schisandrin B), gomisin A and gomisin N(11). Among these constituents, the schisandrin C and schisandrin B are most investigated for their therapeutical potentials for liver diseases.

Diphenyl dimethyl bicarboxylate (DDB), or full name dimethyl-4,4’-bimethyloxy-5,6,5’,6’-dimethylene-dioxydiphenyl-2,2’-bicarboxylate (DDB) (Fig. 1b), is a synthesized intermediate derivative of schizandrin C, an active component isolated from the fructus schizandrae(1, 12). Schisandrin B (Sch B) is another active dibenzocyclooctadiene derivative isolated from the fruit of schisandra chinensis. Compared with Sch B, DDB lacks the cyclooctadiene ring (Fig 1c).

Based on the molecular structure, there are other acronyms for DDB used in the literatures, biphenyl dimethyl-dicarboxylate (BDD)(13, 14), diphenyl dimethyl dicarboxylate (PMC)(15-17).

### DDB for liver disease therapy

DDB is used in the treatment of chronic viral hepatitis for improving liver functions, DDB and Sch B could reduce serum ALT activity in animal models and in humans, it has also antioxidant action for scavenging free radicals and inhibiting lipid peroxidation reaction in in vitro systems(18-20).

### Improving liver function

Clinical study showed that DDB is effective in lowering the serum ALT in patients with chronic viral hepatitis(8) and in protecting against carbon tetrachloride (CCl_4_), D-galactosamine, and thioacetamide induced hepatic damage(21, 22).

In a controlled trial of chronic viral hepatitis, patients who received schisandra extract showed normalized serum ALT levels after 4 weeks, after withdrawal of schisandra treatment ALT remained at normal levels, improvement of other liver function parameters was less pronounced(11). Schisandra was effective in relieving symptoms of sleeplessness, fatigue, abdominal tension and diarrhea(11). This was confirmed by another randomized controlled clinical trial which showed DDB significantly improved liver functions in patients with hepatitis B, by lowering serum ALT, bilirubin, a-fetoprotein, and alleviating symptoms(8). Other researchers found that DDB can effectively normalizes elevated ALT levels in patients with chronic liver diseases of different etiologies, the aspartate aminotransferase (AST), gamma-glutamyl transferase, and glutamate dehydrogenase levels are not affected, however, after DDB treatment, ALT relapsed in all patients within 2–6 weeks(23).

DDB can improve liver functions of hepatitis B, lower blood bilirubin and a-fetoprotein and alleviate symptoms of patients(4, 5, 24). During the administration of DDB, side effects have not been described previously(23).

The serum AST, ALT are commonly used enzymatic markers in assessment of liver functions(25). In hepatocelluar damage, these enzymes which normally locate in the cytosol are released into the blood circulation. Measurements of these enzymes in blood help evaluate the extent and type of hepatocellular damage(26). In the in vitro liver cell injury experiments, cultured hepatocytes treated with DDB had significant decrease of cellular ALT, indicating that the DDB affects the synthesis and/or degradation of ALT in liver cells(12, 23, 27, 28). It is considered that the normalization of only ALT by DDB treatment does not indicate therapeutic efficacy, therefore DDB is not recommended for the routine treatment of chronic liver diseases(23), especially in the anti-hepatitis B therapy during which the ALT levels is only a monitoring marker of anti-viral efficacy.

### Anti-HBV

In the treatment of viral hepatitis, DDB is mostly indicated as an adjuvant hepatoprotectant. However, recent studies showed it may have effect of anti-HBV. DDB can stimulate Jak/Stat signaling, and induce the expression of interferon alpha (IFN-α) stimulated genes, most notably 6-16 and ISG12(29). When DDB is administered combined with amantadine, it can directly inhibit IFN- α signaling-mediated replication of HBV in infected hepatocytes, this represents a novel treatment potential for chronic hepatitis B(29).

However, in another study, patients with chronic hepatitis C, B, or steatohepatitis, with persistently elevated ALT were treated with DDB, ALT can rapidly normalized in most of the patients and remained normal during treatment. But there was no significant effect on the hepatitis B virus DNA level. Histological examination revealed no improvement on the grade and stage of liver disease(23).

### Treatment of chemicals or drugs induced hepatic injury

DDB is effective in treating or preventing chronic hepatitis induced by chemicals or drug poisoning(22), hepatoprotective effects of DDB were reported against a variety of toxicants(8, 30, 31).

It was found that DDB could protect against carbon tetrachloride (CCl_4_) induced liver damage in mice without obvious side effects(32). DDB also alleviates liver injury induced by D-galactosamine, thioacetamide and prednisolone in animal models(12, 15, 22). DDB inhibits tamoxifen-induced hepatic injury, by reducing the oxidative stress, such as lipid peroxidation, and enhancing the antioxidant enzyme activities. DDB can mediate its biochemical effects through the enhancement of the antioxidant enzyme activities and reduced glutathione level as well as decreasing the level of lipid peroxides(7).

In the concanavalin A (Con A) induced liver injury mice, DDB significantly inhibites the elevation of serum ALT, TBIL and total bile acid levels, but it has no effect on AST(3). The early DNA fragmentation and cell apoptosis was observed in the Con A hepatitis model, the DNA fragmentation and cell apoptosis was considered induced in part by tumor necrosis factor-α (TNF-α)(33), DDB strongly down-regulated expression of TNF-α mRNA(3), this was evidenced by the marked decrease of serum TNF-α level(3). These results indicate that DDB could directly protect hepatocyte DNA from oxidative damage, and inhibit TNF-α mRNA expression in liver tissue, thus prevent the liver damages induced by Con A(3).

DDB is hepatoprotective against erythromycin toxicity in rats. Oral daily administration of toxic dose of erythromycin stearate (100 mg/kg body weight) was given to male rats for fourteen days to induce hepatotoxicity. The results of DDB treatment showed that DDB (100 mg/kg body weight) significantly prevented the erythromycin stearate induced liver damage. The biochemical parameters(ALT, AST, TBIL, total lipids and cholesterol) and histology improved compared with ursodesoxycholic acid or Silymarin, as reference drugs(34).

The mechanisms of DDB hepatoprotection might involve the follows. The first, DDB has hepatoprotective effect and functions as a potent antioxidant agent when it is used in the treatment of viral and chemically induced hepatitis(27, 28, 35). The second, pharmacological study shows that DDB increases liver protein and glycogen synthesis and has an inducing effect on the cytochrome P-450 enzyme system(36), therefore DDB has the effects of anti-toxic, anti-carcinogenic and anti-mutagenic effects(36). The third, DDB may protect hepatocytes by stimulating the hepatic mitochondrial reduced glutathione (GSH) antioxidant system via activation of GSH related enzyme, this is evidenced by the increased tissue GSH(37). GSH plays an important role in numerous cellular functions, including DNA synthesis, regulation of cytosolic Ca^2+^ homeostasis, and detoxification of reactive oxygen species(38-40). Deficiency of cellular GSH increases prooxidant production, and enhances apoptosis(10, 41). GSH works with the antioxidant enzymes, such as Se-glutathione peroxidase, glutathione S-transferases, and glutathione reductase, in combating reactive oxygen species and maintaining cellular glutathione status, in this process, the maintenance of mitochondrial glutathione status was critical for cell survival(42, 43).

### Immunological modulation

When mice were administered 20% ethanol alone, splenic plaque forming cells and hemaglutination titers to sheep red blood cells, and the secondary IgG antibody response to bovine serum albumin were decreased, however, they restored to normal level after DDB treatment. The elevations of serum ALT and total protein levels caused by ethanol were also reduced to normal by the treatment of DDB. The lowered serum albumin and albumin: globulin ratio were also increased to normal level. These findings indicate that DDB has a protective effect against ethanol induced humoral immunosuppression(16, 44).

DDB also can restore the cellular immune functions suppressed by CCl_4_ or ketoconazole in mice(15, 17), indicated by the natural killer cells and phagocytic activity were significantly augmented(45). These results showed DDB restores and prevents the immune functions depression by hepatotoxicity agents.

### Treatment of malignancy

DDB has anticancer activity and inhibits malignancy differentiation effects on cancer cells(46). In an in vitro study, DDB was confirmed to have multidrug resistance chemosensitizing effect on a panel of cancer cell lines. DDB at nontoxic concentrations partly reversed the resistance to vincristine, doxorubicin, paclitaxel in acquired multidrug resistance breast carcinoma MCF-7/Adr cells, KBv200 and intrinsic multidrug resistance human hepatocarcinoma Bel(7402) cells, confirmed by increasing the intracellular accumulation of doxorubicin and inhibited surface P-gp expression in MCF-7/Adr cells. Also DDB promoted doxorubicin-induced apoptosis of Bel(7402) cells by enhancing caspase-3 activation. These results indicate that DDB has multidrug resistance reversal activity by inhibiting P-gp when used combined with cancer drugs (47).

DDB can prevent malignant transformation of WB-F344 rat liver epithelial cells induced by 3-methylcholanthrene and 12-O-tetradecanoyl phorbol 13-acetate. This showed DDB has a potential chemopreventive effect on hepato-carcinogenesis induced by carcinogens in vitro(48).

### Schisandrin B versus DDB

In addition to DDB, schisandrin B (Sch B) is another main active component from the schisandra chinensis. It was indicated that the Sch B pretreatment independently enhances glutathione antioxidant status (mtGAS) and induces heat shock protein (HSP) 25/70 (HSP 25/70) production, particularly under conditions of oxidative stress, therefore protecting against CCl_4_ hepatotoxicity in mice(49) and against TNF-α induced hepatic apoptosis in D-galactosamine-sensitized mice(50), whereas the DDB did not have this effect(49). Sch B protects against CCl_4_ induced hepatotoxicity(37), myocardical ischemia/reperfusion injury and brain oxidative damage in rodents(51, 52). These effects are considered attributing to the enhancement of cellular glutathione antioxidant status(37, 51, 52), particularly in the mitochondrion(53). However, the DDB did not stimulate mitochondrial glutathione status nor did protect CCl_4_ induced hepatotoxicity in mice(28).

In another study, pretreating mice with Sch B or DDB significantly lowers the CCl_4_ induced serum ALT in mice, with the inhibitory effect of Sch B being much more potent. Sch B, but not DDB, could decrease the serum sorbital dehydrogenase (SDH) activity. The lowering of serum SDH activity, indicative of hepatoprotection against CCl_4_ toxicity, by Sch B treatment was associated with an enhancement in hepatic mitochondrial glutathione redox status as well as an increase in mitochondrial glutathione reductase (mtGRD) activity in CCl_4_-treated mice. Again, DDB pretreatment, though enhancing both hepatic mitochondrial glutathione redox status and mtGRD activity in control animals, did not produce any beneficial effect in CCl_4_ treated mice. These differences in hepatoprotective action against CCl_4_ toxicity between Sch B and DDB might therefore be related to their ability to maintain hepatic mitochondrial glutathione redox status under oxidative stress condition(28).

All these apparent differences and discrepancies regarding to the efficacy of DDB from the aforementioned might also result from the different research protocols, differences of individual laboratories, or even the uncertainties of DDB effects under different scenarios.

The fact that Sch B and DDB may share the same efficacy in the treatment of hepatitis is not surprising, since both compounds are derived from the fruits of schisandra chinensis with the similar molecular structure.

## Conclusions

The leakage of hepatic enzymes such as ALT and AST is commonly used as an indirect index of hepatocellular damage. Numerous studies demonstrated that DDB could lower the blood ALT, it even may not affect the AST levels. Now, the controversy of DDB in treating viral hepatitis is whether DDB can improve liver histology, and whether DDB has any anti-viral effect. Solely lowering blood ALT without improving liver histology is considered no substantial treatment efficacy; furthermore, ALT normalization sometimes occurs only during the DDB treatment period. Normalization of ALT may comfort patients temporarily, and sometimes may lessen the patients’ anxieties. However, in this regard, patients may misunderstand or be misled by this superficial effect, any claims of treatment effect judged by the solely ALT decrease are unethical. Another concern is that during antiviral agent is being used, such as the interferon, nucleotides, the fluctuation of serum ALT is the assessment marker of antiviral efficacy, in these cases, ALT lowering drugs as DDB are not recommended.

There are still too much unknowns about the DDB effects, more randomized controlled trials should be carried out to ascertain these effects. Prior to the full elucidation of the total panel of DDB therapeutical potentials, we should bear in mind that a single drug (herb or compound derived) can not have unlimited therapeutical potentials, viral hepatitis, as a complicated clinical setting, can not be cured by a single drug.

## Abbreviations

DDB: dimethyl diphenyl bicarboxylate
Sch B: schisandrin B
ALT: alanine aminotransferase
AST: aspartate aminotransferase
TBIL: total bilirubin
CCl_4_: carbon tetrachloride
GSH: reduced glutathione
mtGRD: mitochondrial glutathione reductase
SDH: sorbitol dehydrogenase
